# Cholate Clearance: Improving the Assessment of Liver Health Compared to Current Liver Tests

**DOI:** 10.1016/j.gastha.2025.100814

**Published:** 2025-09-18

**Authors:** James R. Burton, Edward Mena, Bhaktasharan Patel

**Affiliations:** Division of Gastroenterology and Hepatology, Anschutz Medical Campus, University of Colorado, Aurora, Colorado; California Liver Research Institute, Pasadena, California; Peak Gastroenterology Associates, PC, Colorado Springs, Colorado

## Pitfalls of Current Liver Function Tests

The current liver function tests are either markers of late-stage disease (bilirubin, albumin, prothrombin time, international normalized ratio (INR), platelet count) or indicators of hepatobiliary injury (aspartate aminotransferase [AST], alanine aminotransferase [ALT], γ-glutamyl transferase [GGT], and alkaline phosphatase [ALP]). Bilirubin, albumin, and INR, which assess liver cell functions, and platelet count, which assesses splenic congestion from portal hypertension, are insensitive, influenced by nonhepatic factors, and may remain in the normal range until very late-stage disease.^1^, ^2^, ^3^

Liver enzyme levels (AST and ALT) in the blood reflect necroinflammation and do not measure liver function or physiology. These “LFTs” do not adequately define or track liver function. For example, patients with acute hepatitis may have extremely elevated levels of AST, ALT, and total bilirubin; patients with bile duct obstruction may have significantly elevated ALP, GGT, and total bilirubin; and all may have preserved hepatic function. Conversely, patients with end-stage liver disease and poor liver function may have normal AST and ALT (the necroinflammation has “burnt out”).^4^

## Using Cholate Clearance to Quantify Liver Function and Physiology

Consider a comparison to the kidney. The prototypical quantitative test of organ function is flow-dependent creatinine clearance for quantifying kidney function. This test is based upon the integrity of the nephron as the functional unit of the kidney. In the steady state, serum creatinine level reflects the functional renal mass because the body’s production of creatinine is relatively constant, the only route of excretion from the body is via the urine, and the daily urine output is relatively constant. With renal disease, the number of functioning nephrons declines, creatinine clearance decreases, and serum creatinine increases.

Cholate clearance by the liver is similarly based upon the integrity of the hepatic acinus, the functional unit of the liver. Cholate’s production by the liver is relatively constant, and its clearance is liver-specific and flow-dependent. In addition, cholate clearance in healthy persons, without liver disease, reflects the rate of cholate uptake by isolated hepatocytes, total hepatic blood flow of healthy persons, and first-pass hepatic extraction as measured in humans and animal models.^5^ These features indicate that cholate clearance is a measure of liver function analogous to creatinine clearance as a measure of kidney function.

Given the analogy to creatinine, why can’t serum cholate be used to quantify its clearance? Unlike creatinine, endogenous cholate undergoes enterohepatic cycling (the cholate secreted into bile is either stored in the gallbladder or enters the small intestine and is then absorbed into the portal circulation). The cholate that is not cleared by the liver spills into the systemic compartment and increases peripheral venous blood levels. This enterohepatic cycling and gallbladder contraction or filling contribute to wide swings in peripheral blood cholate levels. Endogenous cholate levels reflect changes in cholate cycling in the enterohepatic circulation rather than hepatic function or physiology. Therefore, if one wishes to use cholate clearance as an indicator of liver health, labeled cholates must be administered and directly measured.

## Measuring Cholate Clearance

We have evaluated 2 methods of evaluating cholate clearance: a dual isotope method involving simultaneous administration of 13C-cholate intravenously and d4-cholate orally (HepQuant SHUNT), and a single isotope method involving only oral administration of d4-cholate (HepQuant DuO). Test compounds are labeled with nonradioactive stable isotopes.

The prototypical test, the dual isotope shunt test, fully quantified the clearance of isotopically labeled cholates.^6^ The test involved simultaneous administration of 13C-cholate intravenously and d4-cholate orally, followed by five blood samples collected over 90 minutes, and concentrations of the administered cholates measured by liquid chromatography-tandem mass spectrometry (LC-MS/MS). The test directly measured the systemic and portal clearances of cholate (ie, hepatic filtration rates [HFRs]). Several test parameters were calculated based on the cholate clearances, including Disease Severity Index (DSI), a score of disease severity from 0 (no disease) to 50 (end-stage disease) based on indexing a patient’s HFRs against maximum HFRs in healthy individuals. These test parameters correlated with stages of fibrosis on liver biopsy, likelihood of varices at endoscopy, development of portal hypertension, improvement after sustained virologic response to hepatitis C virus treatment, and risk for future clinical outcome.

The oral cholate challenge test is a major simplification of the dual cholate shunt test, requiring only an oral dose of d4-cholate followed by 2 blood samples at 20 and 60 minutes.^7^^,^^8^ The oral cholate challenge test is reproducible,^9^ and the results are equivalent to those from the dual cholate shunt test.^10^ The oral cholate challenge test is now available for clinical use and can assist in informing decisions to forgo or perform an endoscopy to check for esophageal varices, as well as determining liver health or degree of impairment. The SHUNT-V Pivotal Study validated DSI from both the dual isotope and oral-only tests with the likelihood of finding large esophageal varices at endoscopy (Table) and demonstrated similar diagnostic performance between test versions.^11^^,^^12^

**Table:** Diagnostic Performance of the DSI ≤18.3 From Cholate Clearance Testing in Ruling out the Presence of Large Esophageal Varices Detected by Endoscopy in the SHUNT-V Study of Subjects With Child-Pugh Class A Cirrhosis (n = 238)^11^

Diagnostic performance (95% CI)	Dual cholate shunt test (V1.1)	Oral cholate challenge test
AUROC	0.82 (0.75–0.88)	0.81 (0.72–0.87)
Sensitivity (%)	100.0 (87.2–100)	96.3 (81.0–99.9)
Specificity (%)	35.1 (28.6–41.9)	39.3 (32.7–46.3)
Positive predictive value (%)	16.5 (11.1–23.0)	16.9 (11.3–23.8)
Negative predictive value (%)	100.0 (95.1–100)	98.8 (93.5–100.0)
Positive likelihood ratio	1.54 (1.39–1.70)	1.59 (1.39–1.81)
Negative likelihood ratio	0.00	0.09 (0.01–0.65)
Missed large esophageal varices (n, %)	0 (0.0%)	1 (3.7%)
Endoscopies prevented (%)	74 (31.1%)	84 (35.3%)

AUROC, area under the receiver operating characteristic curve; CI, confidence interval.

## Cholate Clearance vs LFTs

We performed a direct comparison of DSI from the oral cholate challenge test with standard LFTs in 455 persons with advanced fibrosis or compensated Child-Pugh Class A cirrhosis (Figure).^12^ The cases were grouped by fibrosis or cirrhosis, and the cirrhosis cases further stratified by absence of varices, presence of small varices, or presence of large varices, among the groups. The results were striking: total bilirubin, albumin, and INR in the persons with advanced fibrosis or compensated Child-Pugh Class A cirrhosis overlapped completely with the results of these tests in healthy controls, and failed to show stepwise worsening from no varices to small or large varices. Platelet counts declined only in a subset of late-stage disease, mainly in those with large esophageal varices. In contrast, DSI distinguished liver patients from healthy controls and demonstrated stepwise worsening from no varices to small varices to large varices.^11^^,^^12^

**Figure:**
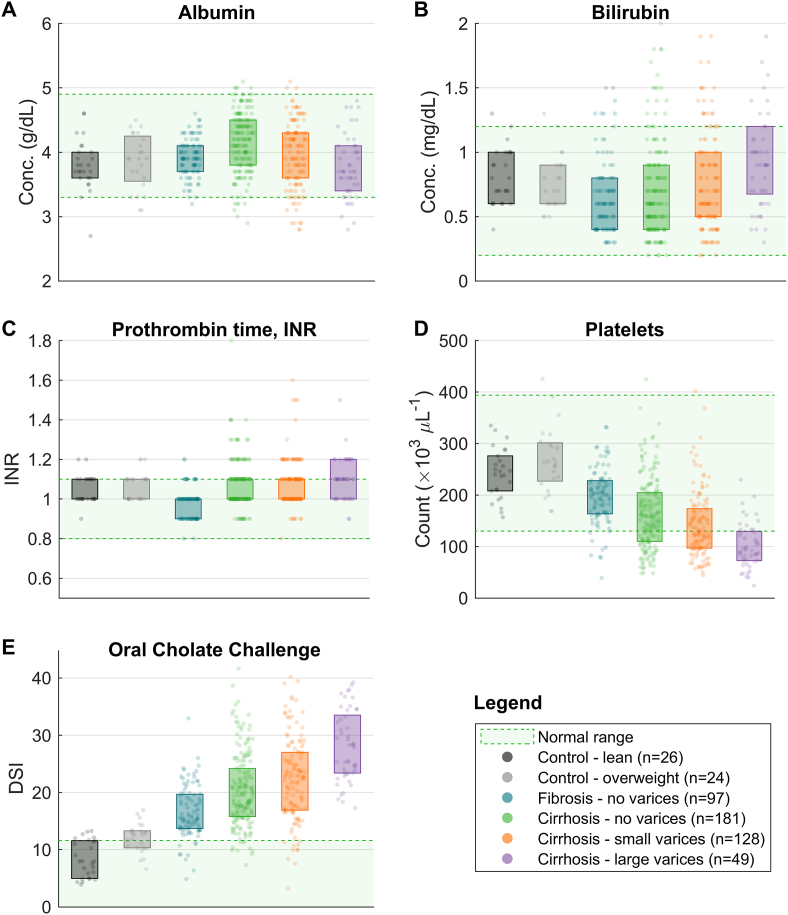
Standard “LFTs” vs the oral cholate challenge test (HepQuant DuO). From left to right, the data encompass healthy controls (n = 26 with lean body mass and n = 24 overweight) and subjects with liver disease of various etiologies and stages (n = 455 in total: n = 97 with fibrosis and no varices, n = 181 with cirrhosis and no varices, n = 128 with cirrhosis and small varices, and n = 49 with cirrhosis and large varices). Each subject is represented by a single marker, and boxes represent the interquartile range.

Currently physicians and other care providers stage a patient’s severity of liver disease based on history, physical examination, laboratory tests, and imaging studies. By providing a continuous quantitative measure of liver health throughout the stages of liver disease, cholate clearance has the potential to improve the assessment of the liver patient not only by hepatologists, but also by gastroenterologists, primary care physicians, endocrinologists, surgeons, and others.

The ability to quantify functional change over the full spectrum of liver disease, as well as over time, can characterize disease severity, monitor progression, or measure therapeutic effects of new compounds. For drug developers, functional assessment may be useful for monitoring the response of the liver to interventional treatments or to injury from an experimental compound, as well as to determine the progression or regression of liver disease over time. Thus, cholate clearance tests can be helpful to inform clinical decision making, as well as to measure efficacy, or lack thereof, in pharmaceutical trials. Each component of these tests bears a distinct relationship to fibrosis stage and functional stages of cirrhosis. Because DSI and the hepatic venous pressure gradient (HVPG) both focus on changes to the portal circulation, DSI may ideally be used as a minimally invasive alternative to the HVPG.^13^ Unlike HVPG, and the Model for End-Stage Liver Disease (MELD) score which assesses the severity of a patient’s liver disease and predicts 3-month mortality rate in patients with cirrhosis, DSI measures hepatic function and impairment over the entire spectrum of liver disease—from F1 (early-stage fibrosis) to F4 (cirrhosis)—and through all the clinical stages of cirrhosis.^14^

## Improving Clinical Decision Making

### Patient 1

A 38-year-old male was admitted with severe alcohol-related hepatitis and hepatic decompensation in early 2023. Physical examination revealed jaundice, ascites and hepatic encephalopathy. He recovered and was discharged from the hospital. Approximately 1 year later, the patient continued to be sober and was working full time. His medications included diuretics, lactulose, and rifaximin. His physical examination was normal with no ascites, peripheral edema, or neurological signs. Laboratory values, noninvasive tests, and clinical scores were total bilirubin 0.6 mg/dL, serum albumin 5.1 g/dL, INR 1.01, platelet count 280,000 μL^-1^, liver stiffness 21.4 kPa, consistent with cirrhosis, and MELD score 6. The oral cholate challenge test was administered and revealed a DSI of 15.2. The latter results indicated low risk for portal hypertension and varices and aided the decision to defer liver transplantation. The results also reinforce the patient’s commitment to alcohol abstinence.

### Patient 2

A 44-year-old male with alcohol-related cirrhosis presented early 2023 with severe alcohol-related hepatitis and hepatic decompensation, complicated by ascites, spontaneous bacterial peritonitis, hepatic encephalopathy, and MELD score of 40. He recovered and was discharged from the hospital. In follow-up 16 months later, the patient remained sober with no liver-related symptoms, no ascites, no peripheral edema, and no neurological signs. Total bilirubin was 2.1 mg/dL, serum albumin 4.1 g/dL, INR 1.4, platelet count 133,000 μL^-1^, and MELD score 12. The oral cholate challenge test was performed and revealed DSI of 38.8, indicating high probability for portal hypertension and large esophageal varices. These results placed him at high risk for future decompensation necessitating close clinical follow-up, and, assuming ongoing abstinence and no further improvement in liver function, the patient may ultimately require liver transplantation.

## Conclusion

The oral cholate challenge test fulfills all the criteria for an ideal test of liver health as set forth in an American Association for the Study of Liver Diseases ‘Emerging Trends’ conference:^15^

(1) Reproducible
(2) Plausibly linked to pathogenesis of disease
(3) Able to assess the whole organ
(4) Minimally invasive and well tolerated
(5) Able to measure effectively all stages of disease, especially early stages
(6) Applicable to relevant populations
(7) Operator independent
(8) Applicable across centers

The test is now available in the clinic for the assessment of patients with chronic liver disease. We anticipate that this quantitative test of liver health will improve patient management by more effectively characterizing disease severity, tracking disease progression, and assessing the impact of treatments. It is our belief that the time has come to move towards more accurate assessment of liver health, the oral cholate challenge test.
